# Active social participation extends the healthy life expectancy of older men without spouses in Japan: The Yamanashi healthy active life expectancy cohort study

**DOI:** 10.1097/MD.0000000000040755

**Published:** 2024-12-06

**Authors:** Takeru Oka, Hiroshi Yokomichi, Zentaro Yamagata

**Affiliations:** a National Center for Geriatrics and Gerontology, Obu City, Aichi, Japan; b Department of Epidemiology and Environmental Medicine, University of Yamanashi, Chuo City, Japan; c The National Center for Child Health and Development, Setagaya-ku, Japan; d The Center for Birth Cohort Studies, University of Yamanashi, Chuo City, Japan.

**Keywords:** cohort analysis, family structure, gender, healthy life expectancy, older adults, social participation

## Abstract

This study aimed to evaluate the association between family structure and healthy life expectancy among older Japanese adults, hypothesizing that social participation increases healthy life expectancy more in older men without a spouse than in older women. This study collected data on Healthy Life Expectancy from 541 older adults between 2003 and 2021 from the Healthy Life Expectancy Study, a cohort study of older adults in Yamanashi Prefecture, Japan. The Japanese long-term care insurance system serves as an indicator of a healthy life expectancy. The family structures of participants were categorized as living alone, with a spouse, and with non-spouse cohabitants, whereas social activity frequency was classified as low or high (Community participation was assessed using a 4-point scale: “often,” “sometimes,” “rarely,” and “never.”). Cox proportional-hazards regression was used to analyze the relationship between participant characteristics and the loss of healthy life expectancy. Each additional year of age for older men and women increases the risk of loss of healthy life expectancy by 13% and 16%, respectively. The risk of loss of healthy life expectancy was higher among older men who lived alone or lived with non-spouse cohabitants than older living with a spouse (hazard ratio [HR]: 1.95, 95% confidence interval [CI]: 1.01–3.75; hazard ratio: 1.66, 95% confidence interval: 1.05–2.64, respectively). However, older men living without a spouse and engaging in high social activity participation had a lower risk of loss of healthy life expectancy than did those with low social activity participation (hazard ratio: 0.35, 95% confidence interval: 0.17–0.71). In conclusion, older men living without a spouse with high social activity participation had longer healthy life expectancies than those living alone with low social activity participation did.

## 1. Introduction

Globally, older adults constitute 16.4% of the population, with older adults in East Asia, Europe, and North America accounting for >25% of the national population. The older adult population in Japan is expected to increase to >35% of the total population by 2050,^[1,2]^ which is a remarkable acceleration compared to the global population and is expected to cause enormous social costs in managing chronic diseases.^[3]^

Various factors contribute to healthy life expectancy, including age, sex,^[4]^economic conditions,^[5,6]^ education,^[7,8]^ social activities,^[9]^ smoking status,^[10]^ lifestyle,^[11]^ and family structure. Over time, changes in family structure have increased the number of older adults living alone in Japan and globally.^[2,12]^ In Japan, older adults living with a spouse have a longer healthy life expectancy than older adults living with persons other than their spouses,^[13]^ where there is an aging of society and a shift to nuclear families.^[1]^ In addition, studies in Japan have shown that older adults living alone have a shorter healthy life expectancy.^[14]^ However, the association between older adults living alone and healthy life expectancy is inconsistent.^[15]^ Older adults who do not live with their spouse tend to be lonely,^[16,17]^ and loneliness shortens the healthy life expectancy.^[18,19]^ However, social activities alleviate loneliness and reduce functional impairments.^[20,21]^ Previous studies have revealed that older men living alone in Asia who received social support had a low risk of functional disability.^[14]^ However, few studies limited to specific geographical areas have linked social activity participation to a reduced risk of functional disability among older men living alone,^[22]^ and there is also a lack of research on older men without a spouse.

This cohort study examined the relationship between healthy life expectancy and family structure among older Japanese adults. Furthermore, the study hypothesized that social participation would increases healthy life expectancy more for older men without a spouse than for older women without a spouse.

## 2. Materials and methods

### 2.1. Participants

Study data were obtained from the cohort data of the Healthy Life Expectancy study investigating older adults living in Yamanashi Prefecture in Japan (Y-HALE).^[23–25]^

**Figure 1.**
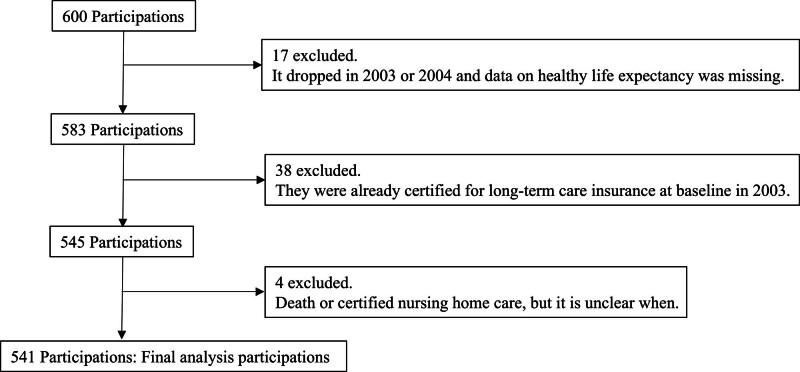
Flowchart of participation.

We enrolled the participants in our cohort in 2 stages. First, we selected 1800 noninstitutionalized older adults (aged ≥ 65 years) who resided in Yamanashi Prefecture between July and August 2002. We visited their homes and, achieve a response rate of 93.3% (1680 participants). Second, we recruited participants for a second survey in September 2003. Among the 782 respondents in October 2003, 600 who agreed to participate were randomly selected and asked more detailed questions.^[23]^ In these 600 participants, 38 were excluded because they were already certified for public long-term care insurance (LTCI) at baseline, 17 dropped out in 2003 or 2004 with missing healthy life expectancy data, and 4 had no documented date of death or certification for public LTCI related to healthy life expectancy. Ultimately, 541 participants were included for in this analysis. Figure 1 illustrates the participant selection process. To collect participants’ baseline data in our cohort, trained researchers visited the participants’ homes and collected information on their sociodemographic characteristics, social activities, diet, psychosocial factors, social capital, and health status using a questionnaire. Data on factors other than outcomes such as functional disability or death, were also collected from the baseline data.

### 2.2. Measurements

#### 2.2.1. Healthy life expectancy

Began (2000) Japan’s public LTCI to assess the “final stage of care” needs. LTCI, a nationally standardized validated measure, classifies older adults with frailty into 7 levels, as shown in Table S1, Supplemental Digital Content, http://links.lww.com/MD/O91 (2 Support Needs levels [1 and 2] and 5 Care Needs levels [1–5]), with higher levels indicating greater care needs. LTCI and its needs assessment and certification system are well accepted in Japan.^[26]^ The it determine an applicant’s eligibility for LTCI benefits according to physical and cognitive functioning using a standardized procedure that includes an interview with a trained professional, physician consultation, computer-based provisional determination, and final decision by the local insurance commission. The LTCI describes a care level of ≥ 2 as a loss of healthy life expectancy^[14]^ because people at this level have difficulty rising from a sitting or lying position or walking and some may require full-time supportive care in their daily lives (Table S1, Supplemental Digital Content, http://links.lww.com/MD/O91).^[24]^ The time to functional disability or death, based on LTCI data for functional disability and mortality, was defined as healthy life expectancy.

#### 2.2.2. Follow-up

Data on deaths and LTCI certifications were collected monthly from 2003 to 2021. Follow-up consultations regarding functional disability or death were obtained annually via mail or telephone interviews. We reminded the participants thrice that is, sending a reminder via a postcard 1 week after the deadline, resending the questionnaire 10 days after the first reminder, and making a phone call 10 days after the second reminder. Participants reported their health status, including their recent eligibility for LTCI (date of eligibility and level of benefits for which they were eligible). For those who died, the family members were asked to report the date and cause of death. Alternative data sources, such as local newspaper obituaries covering deaths reported by the participant’s family, were used to validate death information. If a participant was found or suspected of having died, the participant’s family was contacted to confirm the accuracy of the information. Responses related to LTCI certification were verified by participant’s care plan directors. Regarding outcomes, responses were collected by sending questionnaires from November to December, with follow-up reminders sent a month later. Therefore, for participants with known data on the year of death or LTCI certification, but without specific monthly information, the date was set to June of the known year and treated as censored.

#### 2.2.3. Family structure

We created a family structure variable with 3 categories: living with a spouse, living alone, and living without a spouse, but with at least 1 non-spouse cohabitant. These categories were based on prior Asian research that classified living with a spouse alone as an isolated category regardless of the presence of other family members.^[14]^ The items for family structure were developed based on the original questionnaire and response options provided in Table S2, Supplemental Digital Content, http://links.lww.com/MD/O91. In the Asian study, older adults who lived with their spouses were included as 1 category because the presence or absence of children showed no significant difference among these older adults.^[13]^ Furthermore, previous studies in Japan revealed that several older adults live with 1 or more people (other than their spouse) who were usually their blood relatives.^[14]^

#### 2.2.4. Covariates

Covariates included individual factors (sex, age, and education),^[4,7]^ economic status,^[5,6]^ health status (presence of illness or disability), lifestyle (smoking status, drinking status, and exercise habits),^[11]^ and social participation (local community participation).^[20]^ Community participation was assessed using a 4-point scale: “often,” “sometimes,” “rarely,” and “never.” The total score for the 8 types of local community (senior citizen club, neighborhood association, hobby, sports, volunteer, religion, festivals and bon dance, and teaching activity) participation status frequencies was divided into 2 categories: high and low participation, based on the median. These items were developed based on the original questionnaire and response options provided in Table S2, Supplemental Digital Content, http://links.lww.com/MD/O91.

### 2.3. Statistical analysis

**Figure 2.**
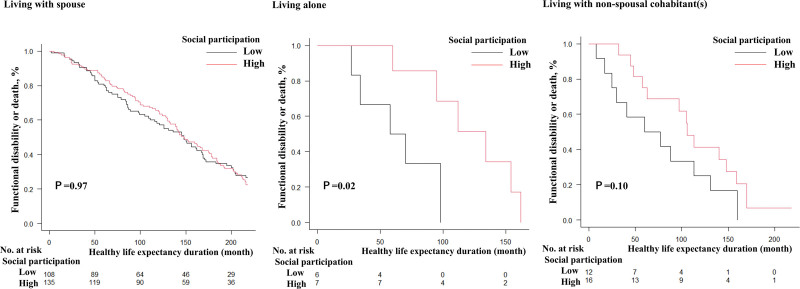
Loss of healthy life (functional disability or death) for social participation by strata of family structure in older men.

**Figure 3.**
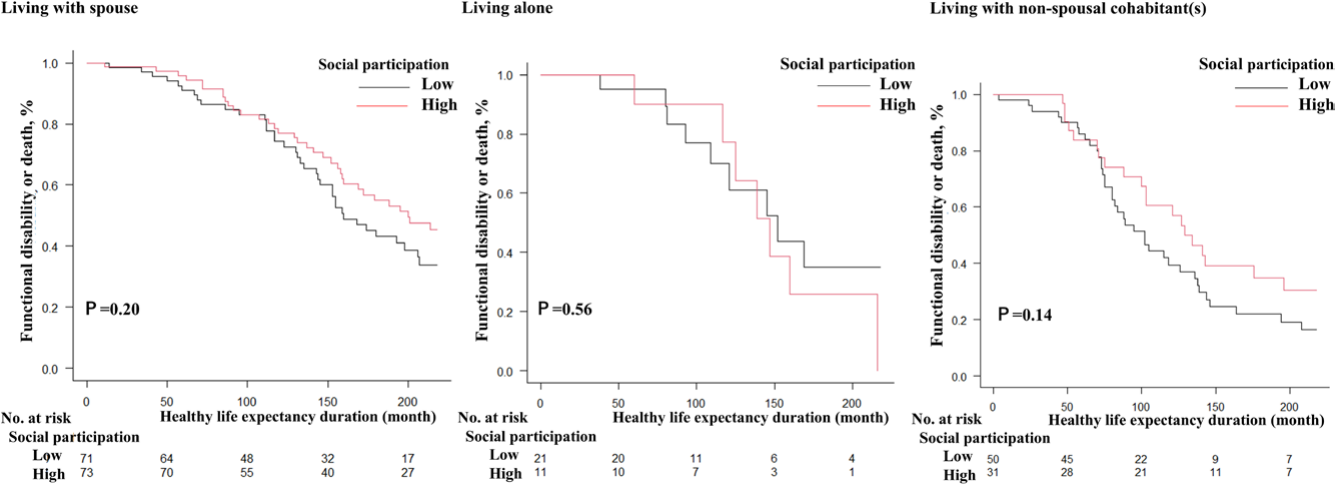
Loss of healthy life (functional disability or death) for social participation by strata of family structure in older women.

The 3 categories of family structure and other covariates were summarized using descriptive statistics. Subsequently, in cases of missing data, all variables were imputed using multiple imputations, with 20 imputations. Proportional hazard regressions in monthly periods were used to describe the relationship between covariates, functional disability, and death (health loss) as outcomes. To test the impact of social participation status on healthy life expectancy, we plotted Kaplan–Meier curves stratified by family structure (Figs. 2 and 3) and conducted inverse probability weighting using logistic regression to create a dataset encompassing all covariates except for social participation, with healthy life expectancy loss as the outcome. We then used this dataset in a proportional hazard regression to evaluate the effect of social participation on healthy life expectancy among older adults with or without a spouse. This analysis stratified participants by the presence or absence of a spouse, rather than by family structure, to consider the heterogeneity in healthy life expectancy loss between men and women with or without a spouse. The proportional hazards assumption was tested using the GLOBAL test. The test was conducted to ensure that the assumption of proportionality was not violated in the Cox proportional hazards models. All statistical analyses were conducted using R software (version 4.3.1; R Foundation for Statistical Computing, Vienna, Austria), utilizing the packages “mice,” “survival,” “aod,” and “car.” Statistical significance was set at *P*-values < 0.05.

### 2.4. Ethics statement

This study was approved by the Ethics Committee of the School of Medicine, University of Yamanashi (approval number: 152) and was conducted in compliance with the ethical guidelines and regulations outlined in the Declaration of Helsinki. Participants provided written, informed consent and were notified that they could withdraw from the study at any time by submitting a written request or making a phone call if they chose to discontinue their participation.

## 3. Results

### 3.1. Participant characteristics

Among the 541 participants in the final analysis, men (n = 284) were more common than women (n = 257) were. Men were more likely to live with their spouses, earn more, attain a higher educational level, have better health, and participate more in social activities than women. By contrast, women were more likely to smoke less, consume less alcohol, and exercise more habitually (Table 1).

**Table 1:** Characteristics of participating older men and women in Japan in 2003 to 2021 (n = 541).

Characteristics	Men	Women
	n (%) n = 284 (53)	n (%) n = 257 (47)
Age, in year (mean (SD))	76.1 (6.7)	76.2 (6.9)
*Family structure*		
Living with spouse	243 (85)	144 (56)
Living alone	13 (5)	32 (13)
Living with non-spousal cohabitant(s)	28 (10)	81 (31)
*Annual income (Missing = 3*)		
<1 million yen	50 (18)	140 (55)
1–2.5 million yen	108 (38)	82 (32)
≥2.5 million yen	125 (44)	33 (13)
*Educational attainment (Missing = 23*)		
Less than high school	152 (56)	158 (65)
High school or higher	122 (44)	86 (35)
*Health status*		
No illness or disability	182 (64)	155 (60)
Has an illness or disability*	102 (36)	102 (40)
*Smoking habit (Missing = 1*)		
Never	73 (26)	243 (95)
Past	148 (52)	7 (3)
Current	63 (22)	6 (2)
*Daily alcohol consumption*		
No	84 (30)	185 (72)
Yes(0 < g ≤ 50)	142 (50)	58 (23)
Yes(50 < g)	58 (20)	14 (5)
*Exercise habitually*		
No	164 (58)	136 (53)
Yes	120 (42)	121 (47)
*Social participation frequency* †		
Low	126 (44)	142 (55)
High	158 (56)	115 (45)

SD = standard deviation.

* Has an illness or disability and is visiting a hospital.

† Social participation frequency (local community participation) was assessed using a 4-point scale: “often,” “sometimes,” “rarely,” and “never.” The total score was divided 2 categories: High and Low, based on the median.

### 3.2. Participant outcomes

The overall median healthy life expectancy (time until the loss of health or death) was 129 months. A total of 348 participants attended to the outcome of interest during the follow-up period. Of these, 194 died, and 154 were classified as needing LTC.

### 3.3. Loss of healthy life expectancy among older adults in Japan

The risk of loss of healthy life expectancy increases with age in both older men and women. Every additional annual increase in both older men and women, increases their risk of loss of healthy life expectancy by 13% and 16%, respectively. The risk of loss of healthy life expectancy was higher among older men who lived alone or lived with non-spouse cohabitants than among those living with a spouse (hazard ratio [HR]: 1.95, 95% confidence interval [CI]: 1.01–3.75 and HR: 1.66, 95% CI: 1.05–2.64, respectively). Older women with high school or higher educational levels had a lower risk of loss of healthy life expectancy than those with a lower educational level (HR: 0.60, 95% CI: 0.40–0.89). The proportional hazards assumption was assessed using the GLOBAL test, which indicated no violation of the assumption. The relationships between family structure and loss of healthy life expectancy among older women and between educational level and loss of healthy life expectancy among older men were not statistically significant (Table 2).

**Table 2:** Hazard ratios of loss of healthy life for men and women in Japan in 2003 to 2021 (n = 541).

Risk factors	Men		Women	
	Crude HR (95% CI)	Adjusted HR† (95% CI)	Crude HR (95% CI)	Adjusted HR† (95% CI)
Age, year	1.14 (1.11–1.17)***	1.13 (1.10–1.16)***	1.15 (1.12–1.18)***	1.16 (1.12–1.20)***
Family structure				
Living with spouse	Ref	Ref	Ref	Ref
Living alone	2.39 (1.29–4.44)**	1.95 (1.01–3.75)*	1.42 (0.83–2.45)	0.66 (0.37–1.18)
Living with non-spousal cohabitants	2.20 (1.43–3.36)***	1.66 (1.05–2.64)*	2.07 (1.46–2.94)***	0.99 (0.67–1.48)
Annual income				
<1 million yen	Ref	Ref	Ref	Ref
1–2.5 million yen	0.64 (0.43–0.95)*	0.83 (0.55–1.25)	0.89 (0.62–1.26)	1.05 (0.73–1.52)
≥2.5 million yen	0.65 (0.45–0.96)*	1.00 (0.66–1.51)	0.44 (0.24–0.81)**	0.57 (0.29–1.11)
Educational attainment				
Less than high school	Ref	Ref	Ref	Ref
High school or higher	0.52 (0.39–0.70)***	0.83 (0.60–1.14)	0.49 (0.34–0.72)***	0.60 (0.40–0.89)*
Health status				
No illness or disability	Ref	Ref	Ref	Ref
Has an illness or disability†	1.51 (1.14–2.00)**	1.09 (0.80–1.47)	1.73 (1.24–2.41)**	1.14 (0.81–1.61)
Smoking habit				
Never	Ref	Ref	Ref	Ref
Past	0.89 (0.64–1.24)	0.74 (0.52–1.05)	1.83 (0.75–4.47)	2.07 (0.77–5.53)
Current	1.04 (0.69–1.54)	0.96 (0.52–1.48)	0.85 (0.27–2.67)	1.51 (0.45–5.00)
Daily alcohol consumption				
No	Ref	Ref	Ref	Ref
Yes(0 < g ≤ 50)	0.86 (0.63–1.18)	1.06 (0.75–1.48)	0.69 (0.46–1.05)	0.65 (0.42–1.01)
Yes (50 < g)	0.54 (0.35–0.82)**	0.83 (0.55–1.25)	0.64 (0.30–1.38)	1.11 (0.47–2.64)
Exercise habitually				
No	Ref	Ref	Ref	Ref
Yes	1.24 (0.94–1.63)	1.02 (0.76–1.38)	0.80 (0.57–1.11)	0.91 (0.65–1.28)
Social participation frequency‡				
Low	Ref	Ref	Ref	Ref
High	0.90 (0.68–1.18)	0.81 (0.61–1.09)	0.70 (0.50–0.99)*	0.92 (0.64–1.31)

CI = confidence interval, HR = hazard ratio.

* *P* < .05.

** *P* < .01.

*** *P* < .001.

† Hazard ratios were calculated in Cox proportional model with adjustment of all explanatory variables.

‡ Social participation frequency (local community participation) was assessed using a 4-point scale: “often,” “sometimes,” “rarely,” and “never.” The total score was divided 2 categories: High and Low, based on the median.

### 3.4. Social participation and loss of healthy life expectancy among older adults in Japan

Older men without a spouse who participated more frequently in social participation had a lower risk of loss of healthy life expectancy than those without a spouse who participated less frequently (HR: 0.35, 95% CI: 0.17–0.71) (Table 3, Fig. 2). The proportional hazards assumption was assessed using the GLOBAL test, which indicated no violation of the assumption. Social participation did not influence the loss of life expectancy among older women regardless of their family structure (Table 3, Fig. 3).

**Table 3:** Hazard ratio of loss of healthy life for social participation by strata of gender and family structure (n = 541).

Adjusted hazard ratio† (95% CI)	Men		Women	
	With spouse	Without spouse	With spouse	Without spouse
Social participation frequency‡				
Low	Ref	Ref	Ref	Ref
High	0.81 (0.60–1.110)	0.35 (0.17–0.71)**	0.81 (0.51–1.30)	0.84 (0.51–1.38)

CI = confidence interval, HR = hazard ratio.

** *P* < .01.

† Adjusted hazard ratios were adjusted for covariates of age, Educational attainment, Annual income, Educational attainment, Health status, Daily alcohol consumption, Exercise habitually.

‡ Social participation frequency (local community participation) was assessed using a 4-point scale: “often,” “sometimes,” “rarely,” and “never.” The total score was divided 2 categories: High and Low, based on the median.

## 4. Discussion

### 4.1. Summary of results

We evaluated whether the family structure of older Japanese adults affected their healthy life expectancy, while considering their social participation and gender differences. Older men have a lower healthy life expectancy than older women. Older men who lived alone or with a non-spousal cohabitant had a decreased healthy life expectancy compared to those who lived with a spouse, but this association was not seen in older women. Older women who were educated to at least a high school level had increased life expectancy. Participation in social activities increases the healthy life expectancy of older men living without spouses.

### 4.2. Family structure and healthy life expectancy

Both men and women living with a non-spouse cohabitant were at a higher risk of loss of healthy life expectancy than those living with a spouse. Similar findings have been observed in previous studies.^[27,28]^ In addition, older adults living only with their children were at a high risk of declining healthy life expectancy in Asia.^[13]^ In the previously mentioned loss of healthy life expectancy, older adults without spouses living with their children promote dependence on them and accelerate the loss of healthy life expectancy.^[29,30]^

### 4.3. Possible reasons for gender differences

Older men without spouses experience several physical and mental health problems.^[31,32]^ Older men who live alone have small social networks, tend not to belong to social activity groups,^[33]^ and are likely to be lonely and socially isolated.^[34]^ These social characteristics may be largely due to differences in the way people of different genders create social relationships. Older men in Japan generally have more social networks with work colleagues, whereas older women have more social networks within their neighborhoods.^[35]^ Women have larger networks and support from multiple sources, whereas men tend to rely solely on their spouses.^[36]^ Thus, older men in Japan are less likely to form new social networks after retirement. Furthermore, loneliness and social isolation among older adults, particularly unmarried men, are directly associated with an increased risk of frailty.^[37,38]^ Moreover, social isolation may cause health-related behaviors related to food intake and smoking.^[39]^

Conversely, women have larger social networks than men,^[36]^ and the absence of a spouse and living alone are not considered to be determinants of loneliness and isolation, even in situations where family relationships centered on spouses are less common.^[15]^A high proportion of Japanese women do housework and childcare while employed, whereas men tend to be more engaged in work.^[40]^ The ability of women to smoothly transition to the role of housework may contribute to an increase in a healthy life expectancy after the loss of a spouse regardless of whether they live alone or with their children. Thus, having multiple roles contributes to long healthy life expectancy.^[41]^

### 4.4. Mechanisms of impact of social participation on healthy Life expectancy

It is consistent that extensive participation in social activities is associated with a reduced risk of developing functional disability in the elderly.^[42,43]^ Social participation has been shown to benefit physical and mental function the older adults through physiological, behavioral, and psychological pathways.^[44]^ First, physiological benefits include reductions in biomarkers of disease risk, such as inflammatory value markers.^[45]^ Second, regular social participation may increase physical activity levels.^[46]^ Third, depending on the activity and other factors, it is likely to be associated with gaining a sense of psychosocial purpose and new opportunities for fulfillment and with active and engaged behavior.^[47,48]^ In addition, they contribute to mental health risk reduction.^[49,50]^ Fourth, it has been found to positively affect health behaviors, such as improved dietary habits, including vegetable intake, and reduced smoking behavior.^[39,51]^

Thus, the effects of social participation on physical and mental functions are manifold and are said to be positive from the perspective of frailty,^[52]^ which is considered a precursor to health loss.^[37,38]^

### 4.5. Implications of social participation to extend healthy life in older men without spouses

Specifically, participation in hobbies and sports groups has been shown to reduce the risk of isolation,^[53]^ improve subjective health views, and reduce functional impairment in the older me.^[54]^ Other studies have shown that spontaneous participation in team sports and activities such as walking and socializing with friends can increase healthy life expectancy.^[55,56]^

Based on the above, it is important to bear in mind the following 2 points: (1) physical activities such as gardening, sports, and walking for older men and (2) providing activities in which older men themselves are interested, while avoiding over-intervention so that they can engage in these activities on their own initiative. It is therefore important to consider these 2 points aspects.

### 4.6. Strengths and limitations

First, this study followed up older adults for >19 years to evaluate their healthy life expectancy. Second, the assessment measures of healthy life expectancy were highly objective and standardized.

This study has some limitations. First, it was conducted in a specific geographical region of Japan; therefore, the results may not be generalizable to other populations. Second, the public insurance system used in Japan to define healthy life expectancy may have underestimated healthy life expectancy in older adults because of its self-assessment system. Third, participant attrition limited the sample size.

## 5. Conclusion

Although older men living without a spouse tend to have shorter healthy life expectancy compared to older women, older men actively participated in social activities exhibited a longer healthy life expectancy compared to other men within this group. This highlights the potential value of encouraging social participation among older men without a spouse.

## Acknowledgments

We would like to express our gratitude to all participants and their families in the Y-HALE cohort and to all public officers in Yamanashi Prefecture, Japan.

## Author contributions

**Conceptualization:** Takeru Oka, Zentaro Yamagata.

**Data curation:** Takeru Oka, Hiroshi Yokomichi, Zentaro Yamagata.

**Formal analysis:** Takeru Oka, Hiroshi Yokomichi, Zentaro Yamagata.

**Funding acquisition:** Takeru Oka.

**Investigation:** Hiroshi Yokomichi, Zentaro Yamagata.

**Methodology:** Takeru Oka, Hiroshi Yokomichi, Zentaro Yamagata.

**Project administration:** Takeru Oka, Hiroshi Yokomichi, Zentaro Yamagata.

**Resources:** Hiroshi Yokomichi, Zentaro Yamagata.

**Software:** Takeru Oka

**Supervision:** Hiroshi Yokomichi, Zentaro Yamagata.

**Validation:** Hiroshi Yokomichi, Zentaro Yamagata.

**Visualization:** Takeru Oka, Hiroshi Yokomichi, Zentaro Yamagata.

**Writing – original draft:** Takeru Oka.

**Writing – review & editing:** Hiroshi Yokomichi, Zentaro Yamagata.

## Supplementary Material


