# Supplementary material for: Active social participation extends the healthy life expectancy of older men without spouses in Japan: The Yamanashi healthy active life expectancy cohort study
Source: Medicine (Baltimore). 2024 Dec 6;103(49):e40755. doi: 10.1097/MD.0000000000040755 (PMC11631014; doi:10.1097/MD.0000000000040755)
Supplement: Supplementary file 1 [file medi-103-e40755-s001.docx]

| **Supplementary Table 1: Definitions of levels used in the Japanese public long-term care insurance system** | | | |
| --- | --- | --- | --- |
|  |  |  |  |
| **Level** | **ADL condition** | **Available services** | **Available monthly cost limit  for service [Japanese yen]** |
| Preventive-1/ Preventive-2 | LTCI is needed for some aspects of daily living, but proper care can improve or maintain ADL | LTCI prevention programs | 49,700/10,400 |
| LTCI-1 | Unstable in rising and gait; partial  support needed in toileting, bathing, etc. | Home-visit care, facility-based services | 165,800 |
| LTCI-2 | Difficulty in rising and gait; partial  or complete support needed in toileting, bathing, etc. | Home-visit care up to 3 times a week, or facility-based services | 194,800 |
| LTCI-3 | Impossible in rise and no gait; Complete support needed in toileting, bathing, dressing, and all other basic ADL | Home-visit care during day  evening, home-visit intensive nursing care, or facility rehabilitation services [1 or 2 service times per day] | 267,500 |
| LTCI-4 | Severe decline in ADL capacity; Complete support needed in toileting, bathing, dressing, and all other basic ADL | Home-visit care during day  evening, home-visit intensive nursing care, or facility rehabilitation services [2 or 3 service times per day] | 306,000 |
| LTCI-5 | Complete support needed in ADL; difficulty with communication | Home-visit care during day  evening, home-visit intensive nursing care, or facility rehabilitation services [3 or 4 service times per day] | 358,300 |
| Japan's public long-term care insurance system (LTCI) | | |  |

**Supplementary Table 2: Questionnaire Items Underlying Variables**

| Original Question (= Q) | Original Answer |
| --- | --- |
| Age, in year |  |
| Q. What is your age | Free-text response |
| Family structure |  |
| Q. Do you have a spouse | 1. Yes |
|  | 2. No |
| Q. What is family structure | 1. Living alone |
|  | 2. Living with spouse only |
|  | 3. Other |
| Annual Income |  |
| Q. Please indicate your annual income level | 1. Less than 1 million yen |
|  | 2. 1 million to less than 2.5 million yen |
|  | 3. 2.5 million to less than 5 million yen |
|  | 4. 5 million to less than 10 million yen |
|  | 5. 10 million yen or more |
| Educational attainment |  |
| Q. Could you tell me about your highest level of education | 1. Elementary school |
|  | 2. Former system junior high school |
|  | 3. Former system girls' high school |
|  | 4. New system junior high school |
|  | 5. High school |
|  | 6. Vocational school |
|  | 7. University |
|  | 8. Others |
| Health status |  |
| Q. How would you describe your current health status | 1. I live a normal life without any significant illness or disability |
|  | 2. I have some illness or disability and require medical visits or rehabilitation, but I am mostly able to manage daily activities on my own and can go out independently. |
|  | 3. I have some illness or disability and can manage most daily activities within the home independently, but I am unable to go out on my own. |
| Smoking habit |  |
| Q. I would like to ask about your smoking habits | 1. I have never (or rarely) smoked |
|  | 2. I used to smoke but do not smoke now |
|  | 3. I currently smoke |
| Daily Alcohol consumption |  |
| Q. Total alcohol consumption per day (in grams) Type and daily amount of alcohol usually consumed (1 go = 180 ml) | Please enter the quantity in appropriate units for each item (e.g., bottles for beer, go for sake, glasses for wine |
| 1.Beer |  |
| Large bottle (633 ml) |  |
| Medium bottle (500 ml) |  |
| Small bottle (334 ml) |  |
| Can (350 ml) |  |
| Can (500 ml) |  |
| 2. Sake (Japanese rice wine) |  |
| 3. Shochu (Japanese distilled spirit) |  |
| 4. Wine |  |
| 5. Whiskey, single |  |
| 6. Whiskey, double |  |
| 7. Brandy |  |
| Exercise habitually |  |
| Q. Do you regularly engage in walking or sports | 1. No |
|  | 2. Yes |
| Social participation frequency |  |
| Q. How frequently do you participate in community activities | 1. Frequently |
| 1. Participation in community events (such as festivals, Bon dances, etc.) | 2. Occasionally |
| 2. Participation in neighborhood association or community association activities | 3. Rarely |
| 3. Senior club activities | 4. Not at all |
| 4. Hobby groups or social gatherings with friends |  |
| 5. Volunteer activities |  |
| 6. Activities to share skills or experiences with others |  |
| 7. Religious activities |  |
| 8. Sports gatherings |  |
